# Risk factors for upper and lower type prolonged postoperative ileus following surgery for Crohn’s disease

**DOI:** 10.1007/s00384-021-03969-9

**Published:** 2021-06-17

**Authors:** Ioannis Pozios, Hendrik Seeliger, Johannes C. Lauscher, Andrea Stroux, Benjamin Weixler, Carsten Kamphues, Katharina Beyer, Martin E. Kreis, Kai S. Lehmann, Claudia Seifarth

**Affiliations:** 1 Department of General and Visceral Surgery, Charité – Universitätsmedizin Berlin, Freie Universität Berlin and Humboldt-Universität Zu Berlin, Hindenburgdamm 30, 12203 Berlin, Germany; 2 Institute of Biometry and Clinical Epidemiology, Charité – Universitätsmedizin Berlin, Freie Universität Berlin and Humboldt-Universität Zu Berlin, Charitéplatz 1, 10117 Berlin, Germany; 3 Berlin Institute of Health, Charité – Universitätsmedizin Berlin, Charitéplatz 1, 10117 Berlin, Germany

**Keywords:** Prolonged postoperative ileus, Crohn’s disease, Ileocecal resection, Upper lower gastrointestinal tract, Risk factors

## Abstract

**Purpose:**

Prolonged postoperative ileus (PPOI) is common after bowel resections, especially in Crohn’s disease (CD). The pathophysiology of PPOI is not fully understood. PPOI could affect only the upper or lower gastrointestinal (GI) tract. The aim of this study was to assess risk factors for diverse types of PPOI, particularly to differentiate PPOI of upper and lower GI tract.

**Methods:**

A retrospective analysis of 163 patients with CD undergoing ileocecal resection from 2015 to 2020 in a single center was performed. PPOI of the upper GI tract was predefined as the presence of vomiting or use of nasogastric tube longer than the third postoperative day. Lower PPOI was predefined as the absence of defecation for more than three days. Independent risk factors were identified by multivariable logistic regression analysis.

**Results:**

Overall incidence of PPOI was 42.7%. PPOI of the upper GI tract was observed in 30.7% and lower PPOI in 20.9% of patients. Independent risk factors for upper PPOI included older age, surgery by a resident surgeon, hand-sewn anastomosis, prolonged opioid analgesia, and reoperation, while for lower PPOI included BMI ≤ 25 kg/m^2^, preoperative anemia, and absence of ileostomy.

**Conclusion:**

This study identified different risk factors for upper and lower PPOI after ileocecal resection in patients with CD. A differentiated upper/lower type approach should be considered in future research and clinical practice. High-risk patients for each type of PPOI should be closely monitored, and modifiable risk factors, such as preoperative anemia and opioids, should be avoided if possible.

## Introduction

Transient inhibition of coordinated bowel motility after abdominal surgery is referred to as postoperative ileus (POI) and is a common condition after bowel resections [1]. Although uncomplicated POI resolves spontaneously 2 to 3 days after surgery, a more severe form, the prolonged POI (PPOI), lasts for more than three days [2, 3]. Delayed postoperative recovery of gastrointestinal (GI) function and resumption of oral intake is associated with prolonged hospital stay and increased costs [4]. Additionally, prolonged recovery following PPOI is accompanied by an increased risk of morbidity and mortality. This furthermore entails increased health cost, considerably more discomfort for patients, and increased efforts by their caregivers [4, 5].

Incidence of PPOI was reported in various cohorts in 10 to 30% of patients after abdominal surgery [6, 7]. PPOI is clinically characterized by nausea, vomiting, distension, intolerance to oral intake, and absence of flatus or stool. This phenomenon, induced by direct manipulation of the gut, is common in abdominal surgery, including bowel resections [7–9]. Although manipulation of the gut is reduced in laparoscopy, PPOI also occurs in minimally invasive surgery [10]. Pathophysiology of PPOI is complex and multifactorial. A neurohumoral response to bowel manipulation and surgical trauma, including intestinal inflammation and systemic inflammatory response, seems to play a key role in its development [8, 11–17]. However, detailed pathophysiological mechanisms underlying PPOI are only partly understood.

Patients with Crohn’s disease (CD) represent a group with an increased perioperative systemic inflammatory response, increased postoperative morbidity, and increased risk to develop POI [18, 19]. As CD frequently involves the ileocecal valve, ileocecal resection (ICR) is an established procedure for these patients. The aim of this study was to investigate and compare different types of PPOI concerning their perioperative risk factors and outcomes in patients undergoing ICR for CD. Based on PPOI clinical symptoms, identifying differences between PPOI of upper and lower GI tract may contribute to further investigation of its pathophysiology, prevention, and perioperative management. A symptom-oriented approach may differentiate the surgical patient with minimal symptoms who await the passage of stool to signal recovery and be discharged from the hospital, and the patient who cannot tolerate oral intake and may need parenteral nutrition.

## Methods

### Patients

A retrospective analysis of 187 consecutive patients undergoing ICR due to CD from January 2015 to May 2020 in a single center was performed. The surgical procedure was performed in a standardized manner according to the standard operating procedures of our department. Patients with additional bowel resections due to CD or other reasons were excluded from the analysis. The choice of anastomosis technique (stapled or hand-sewn) was at the discretion of the surgeon. All patients received neostigmine postoperatively until the first stool passage. The study protocol was approved by the Medical Ethical Committee of Charité — Universitätsmedizin Berlin (EA2/136/20).

### Analysis

In this retrospective analysis, data were collected from the hospital's electronic health records system. The analysis included age, gender, body mass index (BMI), ASA (American Society of Anesthesiologists) score, other systemic pre-existing diseases, immunosuppression, preoperative hemoglobin value, preoperative ileus, the experience of the surgeon, type of anastomosis, surgical technique, intraoperative bowel diversion, postoperative use of opioids, peridural anesthesia, postoperative use of neostigmine, 30-day complication rate (Clavien-Dindo classification including anastomotic leak and reoperation) [20], and length of hospital stay. The primary outcome parameter was the incidence of different types of PPOI. Statistical analysis was performed to identify independent risk factors for overall, upper, and lower PPOI and investigate the incidence and risk factors for recurrent and primary PPOI in patients with CD undergoing ICR.

### Definitions

Definitions of POI and PPOI are inconsistent, and both contain different information about the duration and various types of POI. Our study used the following terminology based on the results of a systematic review of Vather et al., which extracted definitions from 52 randomized trials published between 1996 and 2011 investigating POI after abdominal surgery [2]. Postoperative ileus (POI), a partly physiological response to surgical trauma, was defined as intolerance to oral intake and absence of stool passage longer than 24 h after surgery. Prolonged POI was predefined if the interval from surgery until oral intake or passage of stool occurred on or after day four postoperatively without prior resolution of POI.

As described in the Clinical Consensus Update [21] and the study of Bragg et al. [22], recurrent POI was noted if vomiting or nasogastric tube (NG) or inability to tolerate oral diet occurs after apparent resolution of the immediate postoperative POI having already achieved total oral intake. A further classification is based on the presence of an apparent cause. A primary PPOI occurs in the absence of any precipitating reason, while a secondary PPOI occurs after a complication (e.g., anastomotic leak) [22, 23].

To further investigate PPOI, we used a type-stratified approach of PPOI and classified PPOI in two categories according to upper or lower GI tract involvement. PPOI of the upper GI tract was predefined as the presence of vomiting or use of NG after the third postoperative day (POD). PPOI of the lower GI tract was predefined as the absence of defecation for more than 3 days. Besides, we documented time to total oral food intake as a parameter for the postoperative course.

#### Statistical analysis

Continuous variables were expressed as median with interquartile range (IQR) and were analyzed using non-parametric tests (Mann–Whitney U test). Categorical variables were expressed as the number of patients and percentages. For group comparisons concerning categorical variables, the chi-square test was used. The influence of perioperative variables on PPOI and its types was evaluated by univariate analysis. The variables that showed in the univariate analysis a relative association with PPOI or its types with a *p* value less than 0.15 were enrolled in a multivariate logistic regression model to adjust for potential confounders and to identify potential independent risk factors. A *p* value of less than 0.05 was considered statistically significant for the purposes of this study. No Bonferroni correction has been performed due to the exploratory character of this investigation. Odds ratios (ORs) were calculated with a 95% confidence interval (CI). The statistical analysis was performed with SPSS Statistics Software 25.0 (IBM, Armonk, NY, USA).

## Results

### Patient characteristics and postoperative course

During 5 years, a total of 187 patients underwent ICR due to Crohn’s disease at our institution. Of these patients, 163 met the study criteria and were included in the analysis having a median age of 36 years (IQR 26–52) and a female predominance (58.3%). Thirty-two patients (19.6%) were overweight (BMI > 25 kg/m^2^) or obese (BMI > 30 kg/m^2^), while 28 patients (17.2%) were underweighted (BMI < 18.5 kg/m^2^). Most patients (79.1%) were ASA II, and 38.7% of all patients suffered preoperatively from anemia (Hb < 12 g/dl). The majority of the patients (60.1%) were under immunosuppressive therapy, including steroids (27.6%), azathioprine (18.4%), monoclonal antibodies (22.1%), 5-ASA (4.9%), or a combination of them. One-quarter of the patients (25.2%) suffered from chronic ileus at the time of operation due to stenosis of the terminal ileum (Table 1).

**Table 1:** Patient characteristics (*n* = 163)

Variable	*n* (%)
Preoperative	
Age, years; median (IQR)	36 (26–52)
Sex	
Male	68 (41.7)
Female	95 (58.3)
BMI, kg/m^2^; median (IQR)	21.7 (19.5–24.5)
< 18.5	28 (17.2)
18.5–25	97 (59.5)
> 25	32 (19.6)
ASA score	
I	17 (10.4)
II	129 (79.1)
III	17 (10.4)
Preoperative Hb, g/dl	
< 12	63 (38.7)
≥ 12	100 (61.3)
Immunosuppression (total)	98 (60.1)
No	65 (39.9)
Steroids	45 (27.6)
5-ASA	8 (4.9)
Azathioprine	30 (18.4)
Monoclonal antibody	36 (22.1)
Preoperative ileus	
No	115 (70.6)
Chronic	41 (25.2)
Acute	7 (4.3)
Intraoperative	
Emergencies	32 (19.6)
Resident surgeon	28 (17.2)
Approach	
Laparoscopic	85 (52.1)
Open	52 (31.9)
Conversion	26 (16.0)
Anastomosis	
Hand-sewn	104 (63.8)
Stapled	49 (30.1)
Ileostomy	52 (31.9)
Postoperative	
Analgesia	
PDA	26 (16.0)
Opioids	
≤ 5 days	76 (46.6)
> 5 days	57 (35.0)
Neostigmine, days; median (IQR)	4 (3–5)
Clavien-Dindo	
I	6 (3.7)
II	22 (13.5)
IIIa	3 (1.8)
IIIb	27 (16.6)
IVa	2 (1.2)
IVb	1 (0.6)
V	0 (0)
Reoperation	30 (18.4)
LOS, days; median (IQR)	8 (6–11)

Data are described as n (%) or median (IQR)

*BMI* body mass index, *ASA* American Society of Anesthesiology, *Hb* hemoglobin, *PDA* peridural anesthesia, *LOS* length of stay, *SD* standard deviation

Because of previous abdominal surgery, an open approach was decided in 31.9% of the cases, while in 16%, conversion to laparotomy was necessary during the operation. Thirty-two patients (19.6%) underwent an emergency operation because of oncoming or acute ileus. An experienced resident surgeon assisted by an attending surgeon performed 17.2% of the procedures, 30.1% of the anastomoses were stapled, and 31.9% of the patients received an ileostomy (Table 1).

**Fig. 1:**
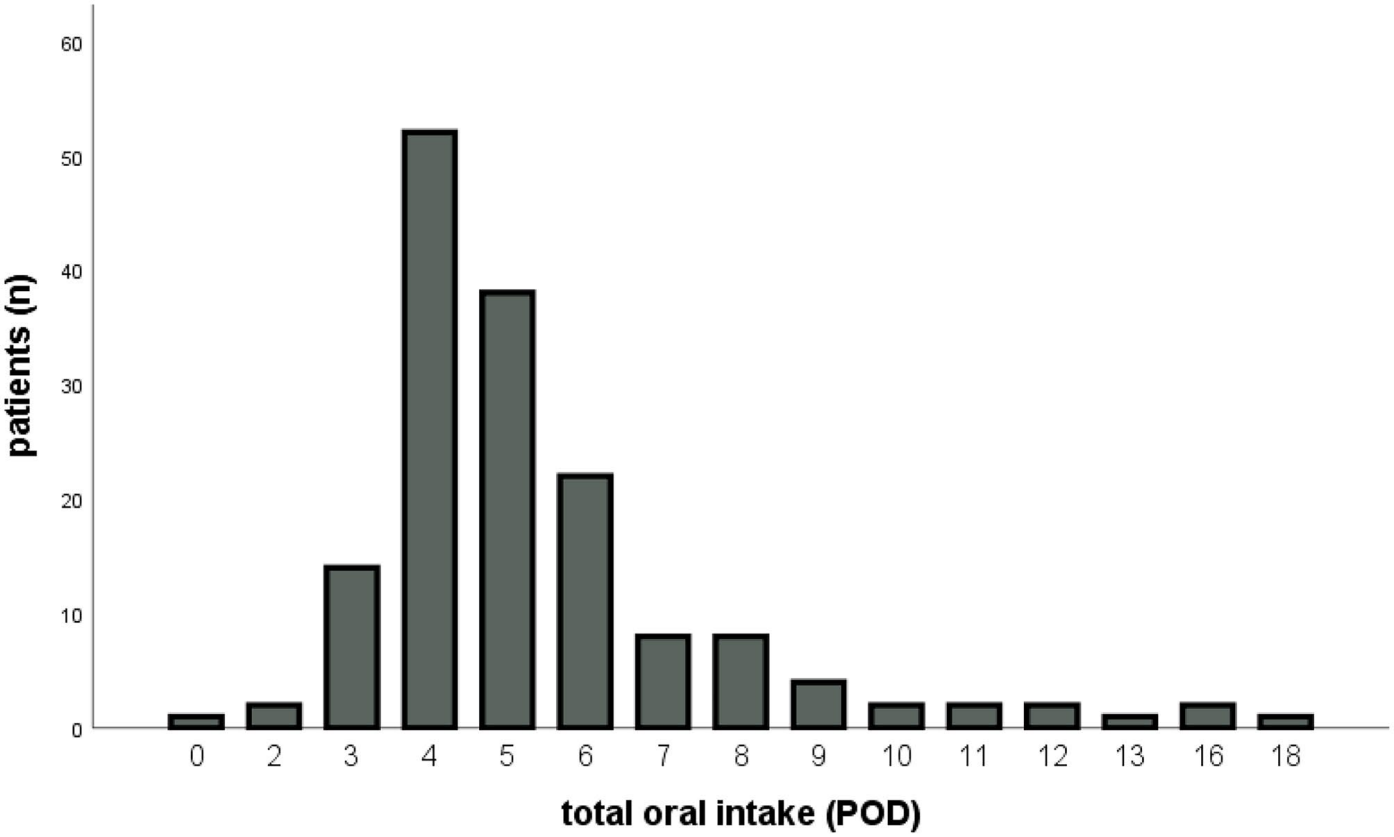
Diagram with the number of patients achieving total oral intake every postoperative day (POD)

**Fig. 2:**
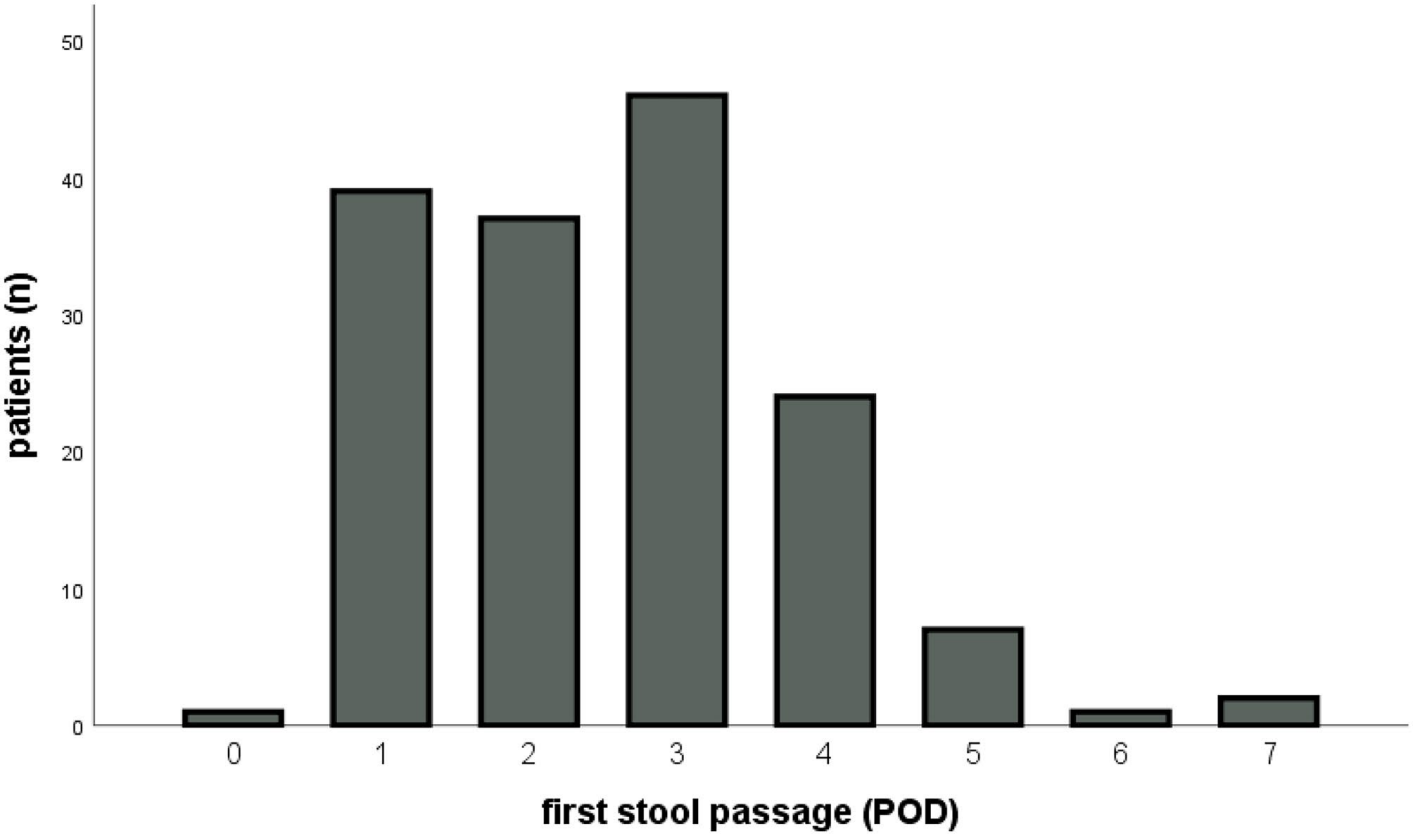
Diagram with the number of patients having first stool passage every postoperative day (POD)

Figure 1 graphically demonstrates the duration to complete oral intake, and Fig. 2 the first stool passage. All patients received neostigmine postoperatively until the first stool passage with a median duration of 4 days (IQR 3–5). Concerning analgesia, we noted 16% of patients having received peridural anesthesia (PDA), while 35% received opioids for more than 5 days. Thirty patients (18.4%) experienced severe postoperative complications (Clavien-Dindo classification IIIb or more) and were reoperated. Twenty-five of them had an anastomotic leak or other surgical site infection, four of them a bleeding and one patient ileus (Table 1). There were no cases of mortality.

### Prolonged POI

A total of 67 patients (42.7%) developed PPOI. In univariate analysis, there was a trend for PPOI to be more frequent in older patients (*p* value 0.051), with preoperative anemia (*p* value 0.117), after hand-sewn anastomosis (*p* value 0.124) and reoperation (*p* value 0.088), but these differences did not reach statistical significance. Ileostomy was significantly less frequent in patients with PPOI (40% vs. 22.4%, *p* value 0.020), while prolonged opioid use after surgery was more often in PPOI patients (26.7% vs. 49.3%, *p* value 0.004). Length of hospital stay was significantly longer in the PPOI group (7 vs. 10 days; *p* value < 0.001) (Table 2). Multivariable analysis identified hand-sewn anastomosis (OR = 2.322; CIs 1.007–5.353; *p* value 0.048), ileostomy (OR = 0.278; CIs 0.117–0.665; *p* value 0.004), prolonged opioid analgesia (OR = 3.740; CIs 1.701–8.223; *p* value 0.001), and reoperation (OR = 3.025; CIs 1.027–8.909; *p* value 0.045) as independent influencing factors for PPOI development (Table 3).

**Table 2:** Comparison of perioperative variables between patients with no PPOI and three different types of PPOI (overall PPOI, primary PPOI, recurrent PPOI)

Variable	No PPOI	Overall PPOI		Primary PPOI		Recurrent POI	
	*n* = 90	*n* = 67	*p* value	*n* = 58	*p* value	*n* = 41	*p* value
Preoperative							
Age, years							
Median (IQR)	34 (25–49)	39 (31–54)	0.051	40 (31 – 56)	0.018	38 (31 – 57)	0.060
Sex							
Male	39 (43.3)	28 (41.8)	0.847	24 (41.4)	0.814	18 (43.9)	0.865
Female	51 (56.7)	39 (58.2)		34 (58.6)		23 (56.1)	
BMI, kg/m^2^							
≤ 25	65 (76.5)	56 (84.8)	0.201	48 (84.2)	0.262	34 (85.0)	0.393
> 25	20 (23.5)	10 (15.2)		9 (15.8)		6 (15.0)	
ASA score							
I–II	83 (93.3)	58 (89.2)	0.375	49 (87.5)	0.237	36 (92.3)	0.867
III–IV	6 (6.7)	7 (10.8)		7 (12.5)		3 (7.7)	
Hb < 12 g/dl	28 (31.1)	29 (43.3)	0.117	27 (46.6)	0.058	13 (31.7)	0.406
Immunosuppression	53 (58.9)	41 (61.2)	0.771	39 (67.2)	0.306	22 (53.7)	0.307
Steroids	28 (31.1)	16 (23.9)	0.318	16 (27.6)	0.647	12 (29.3)	0.873
Azathioprine	14 (15.6)	15 (22.4)	0.275	14 (24.1)	0.193	7 (17.1)	0.733
Monocl. antibody	22 (24.4)	12 (18.2)	0.349	11 (19.3)	0.466	7 (17.1)	0.363
Preoperative ileus	25 (27.8)	22 (32.8)	0.494	21 (36.2)	0.279	15 (36.6)	0.300
Intraoperative							
Emergency	17 (18.9)	14 (20.9)	0.755	13 (8.3)	0.603	7 (17.1)	0.649
Resident surgeon	14 (15.6)	14 (20.9)	0.387	11 (19.0)	0.589	12 (29.3)	0.023
Approach							
Laparoscopic	46 (51.1)	36 (53.7)	0.745	29 (50.0)	0.895	26 (63.4)	0.095
Open	44 (48.9)	31 (46.3)		29 (50.0)		15 (36.6)	
Anastomosis							
Hand-sewn	55 (64.0)	47 (75.8)	0.124	43 (81.1)	0.031	30 (75.0)	0.298
Stapled	31 (36.0)	15 (24.2)		10 (18.9)		10 (25.0)	
Ileostomy	36 (40.0)	15 (22.4)	0.020	15 (25.9)	0.077	14 (34.1)	0.742
Postoperative							
Analgesia							
PDA	16 (18.4)	9 (13.6)	0.431	7 (12.3)	0.328	6 (15.0)	0.727
Opioids > 5 days	24 (26.7)	33 (49.3)	0.004	29 (50.0)	0.004	20 (48.8)	0.045
Clavien-Dindo > IIIb	13 (14.4)	15 (22.4)	0.198	8 (13.8)	0.912	10 (24.4)	0.240
Reoperation	12 (13.3)	16 (23.9)	0.088	8 (13.8)	0.936	12 (29.3)	0.023
LOS, days							
Median (IQR)	7 (6–9)	10 (8–14)	< 0.001	9 (7–12)	0.005	11 (9–19)	< 0.001

Data are described as n (%) or median (IQR)

*BMI* body mass index, *ASA* American Society of Anesthesiology, *Hb* hemoglobin, *PDA* peridural anesthesia, *LOS* length of stay, *SD* standard deviation

**Table 3:** Risk factors for overall, primary and recurrent PPOI by multivariate analysis

Variable	Overall PPOI			Primary PPOI			Recurrent POI		
	OR	95% CIs	*p* value	OR	95% CIs	*p* value	OR	95% CIs	*p* value
Age (older)	1.021	0.998–1.045	0.074	1.021	0.998–1.045	0.072	1.034	1.008–1.060	0.010
Hb < 12 g/dl	2.151	0.977–4.738	0.057	2.090	0.978–4.465	0.057			
Resident surgeon							3.209	1.274–8.088	0.013
Open approach							0.450	0.201–1.010	0.053
Hand-sewn anastomosis	2.322	1.007–5.353	0.048	2.823	1.196–6.664	0.018			
Ileostomy	0.278	0.117–0.665	0.004	0.396	0.168–0.933	0.034			
Opioids > 5 days	3.740	1.701–8.223	0.001	3.406	1.578–7.355	0.002	2.160	0.991–4.707	0.053
Reoperation	3.025	1.027–8.909	0.045				2.896	1.119–7.495	0.028

*OR* odds ratio, *CIs* confidence intervals, *Hb* hemoglobin

### Primary PPOI

We further classified the PPOI patients based on the presence of an underlying cause according to Clinical Consensus Update [21]. Nine of the 67 patients who developed a PPOI (5.8% of all patients) underwent redo surgery and therefore were classified as a secondary PPOI. Consequently, 58 patients (36.9% of all patients) developed a PPOI in the absence of any precipitating cause having a primary PPOI. In univariate analysis, older age (*p* value 0.018), hand-sewn anastomosis (*p* value 0.031), and prolonged opioid use (*p* value 0.004) were significantly correlated to primary PPOI. Preoperative anemia (*p* value 0.058) and ileostomy (*p* value 0.077) showed tendential associations with primary PPOI, but they did not reach significance. Length of stay was significantly longer in the primary PPOI group (7 vs. 9 days; *p* value 0.005) (Table 2). Using multivariable regression analysis for the variables above, three independent factors influencing incidence of primary PPOI were identified: hand-sewn anastomosis (OR = 2.823; CIs 1.196–6.664; *p* value 0.018), ileostomy (OR = 0.396; CIs 0.168–0.933; *p* value 0.034), and prolonged postoperative opioid analgesia (OR = 3.406; CIs 1.578–7.355; *p* value 0.002) (Table 3).

### Recurrent PPOI

Recurrent POI was noted in 25.8% of the patients (n = 41). Twelve of them (29.3%) underwent a reoperation, which may indicate a surgical complication as possible cause for recurrent PPOI. In univariate analysis, factors such as a resident surgeon were operating (*p* value 0.023), and extended postoperative opioid analgesia (*p* value 0.045) and reoperation (*p* value 0.023) were shown to increase POI recurrence. Length of hospital stay was also significantly longer for the patients with recurrent POI (7 vs. 11 days; *p* value < 0.001) (Table 2). Age (*p* value 0.060), open approach (*p* value 0.095), and the variables above were enrolled in a multivariable logistic regression model. Older age (OR = 1.034; CIs 1.008–1.060; *p* value 0.010), resident surgeon (OR = 3.209; CIs 1.274–8.088; *p* value 0.013), and reoperation (OR = 2.896; CIs 1.119–7.495; *p* value 0.028) were independent risk factors for recurrent POI (Table 3).

### PPOI of the upper GI tract

To further analyze the PPOI based on its clinical symptoms, assessment of PPOI for the upper and lower GI tract was performed separately. Incidence of upper PPOI was 30.7%. In initial univariate analysis, older age (*p* value 0.037), prolonged opioid use (*p* value 0.012), severe complications (Clavien-Dindo classification ≥ IIIb; *p* value 0.020), and reoperation (*p* value 0.005) were significantly associated with upper PPOI. Length of hospital stay was also significantly longer for the patients with upper PPOI (7 vs. 11 days; *p* value < 0.001) (Table 4). Multivariable logistic regression suggested older age (OR = 1.026; CIs 1.002–1.051; *p* value 0.037), resident surgeon (OR = 2.822; CIs 1.109–7.176; *p* value 0.029), hand-sewn anastomosis (OR = 3.099; CIs 1.198–8.015; *p* value 0.020), longer postoperative use of opioids (OR = 2.655; CIs 1.212–5.819; *p* value 0.015), and reoperation (OR = 4.890; CIs 1.670–14.319; *p* value 0.004), as independent risk factors for upper PPOI development (Table 5).

**Table 4:** Comparison of variables between patients with no PPOI and PPOI of the upper and lower GI tract

Variable	PPOI upper GI			PPOI lower GI		
	No	Yes	*p* value	No	Yes	*p* value
	*n* = 109	*n* = 50		*n* = 123	*n* = 34	
Preoperative						
Age, years						
Median (IQR)	35 (25–49)	40 (32–56)	0.037	36 (26–51)	37 (25–52)	0.908
Sex						
Male	47 (43.1)	21 (42.0)	0.895	55 (44.7)	12 (35.3)	0.326
Female	62 (56.9)	29 (58.0)		68 (55.3)	22 (64.7)	
BMI, kg/m^2^						
≤ 25	84 (80.8)	39 (79.6)	0.864	88 (75.2)	33 (97.1)	0.005
> 25	20 (19.2)	10 (20.4)		29 (24.8)	1 (2.9)	
ASA score						
I–II	100 (92.6)	43 (89.6)	0.530	110 (91.7)	31 (91.2)	0.928
III–IV	8 (7.4)	5 (10.4)		10 (8.3)	3 (8.8)	
Hb < 12 g/dl	41 (37.6)	18 (36.0)	0.845	38 (30.9)	19 (55.9)	0.007
Immunosuppression	66 (60.6)	30 (60.0)	0.947	74 (60.2)	20 (58.8)	0.888
Steroids	31 (28.4)	14 (28.0)	0.954	39 (31.7)	5 (14.7)	0.051
Azathioprine	19 (17.4)	11 (22.9)	0.494	21 (17.1)	8 (23.5)	0.391
Monocl. antibody	28 (25.7)	7 (14.3)	0.110	27 (22.0)	7 (21.2)	0.927
Preoperative ileus	33 (30.3)	15 (30.0)	0.972	35 (28.5)	12 (35.3)	0.441
Intraoperative						
Emergency	21 (19.3)	10 (20.0)	0.914	23 (18.7)	8 (23.5)	0.531
Resident surgeon	15 (13.8)	13 (26.0)	0.060	23 (18.7)	5 (14.7)	0.590
Approach						
Laparoscopic	58 (53.2)	25 (50.0)	0.707	63 (51.2)	19 (55.9)	0.630
Open	51 (46.8)	25 (50.0)		60 (48.8)	15 (44.1)	
Anastomosis						
Hand-sewn	65 (63.7)	37 (78.7)	0.067	79 (67.5)	23 (74.2)	0.475
Stapled	37 (36.3)	10 (21.3)		38 (32.5)	8 (25.8)	
Ileostomy	37 (33.9)	14 (28.0)	0.456	48 (39.0)	3 (8.8)	0.001
Postoperative						
Analgesia						
PDA	19 (17.9)	7 (14.3)	0.573	22 (18.5)	3 (8.8)	0.179
Opioids > 5 days	32 (29.4)	25 (50.0)	0.012	43 (35.0)	14 (41.2)	0.505
Clavien-Dindo ≥ IIIb	14 (12.8)	14 (28.0)	0.020	21 (17.1)	7 (20.6)	0.636
Reoperation	13 (11.9)	15 (30.0)	0.005	22 (17.9)	6 (17.6)	0.974
LOS, days						
Median (IQR)	7 (6–9)	11 (9–17)	< 0.001	8 (6–11)	9 (6–14)	0.245

Data are described as n (%) or median (IQR)

*BMI* body mass index, *ASA* American Society of Anesthesiology, *Hb* hemoglobin, *PDA* peridural anesthesia, *LOS* length of stay, *SD* standard deviation

**Table 5:** Separate multivariable logistic regression model for PPOI of the upper and lower GI tract

Variable	PPOI upper GI			PPOI lower GI		
	OR	95% CIs	*p* value	OR	95% CIs	*p* value
Age (older)	1.026	1.002–1.051	0.037			
BMI ≤ 25 kg/m^2^				11.653	1.471–92.310	0.020
Hb < 12 g/dl				2.943	1.202–7.203	0.018
Steroids				0.530	0.172–1.627	0.267
Monocl. antibody	0.498	0.181–1.367	0.176			
Resident surgeon	2.822	1.109–7.176	0.029			
Hand-sewn anastomosis	3.099	1.198–8.015	0.020			
Ileostomy				0.123	0.034–0.453	0.002
Opioids > 5 days	2.655	1.212–5.819	0.015			
Reoperation	4.890	1.670–14.319	0.004			

*OR* odds ratio, *CIs* confidence intervals, *BMI* body mass index, *Hb* hemoglobin

### PPOI of the lower GI tract

Incidence of lower PPOI was 20.9%. In univariable analysis, lower PPOI was more frequent in patients with BMI ≤ 25 kg/m^2^ (*p* value 0.005), preoperative anemia (*p* value 0.007), and ileostomy (*p* value 0.001). Preoperative steroid therapy was tendentially associated with a decreased risk of lower PPOI (*p* value 0.051). Length of hospital stay was not significantly longer in patients with lower PPOI (8 vs. 9 days; *p* value 0.245) (Table 4). A multivariable logistic regression model suggested BMI ≤ 25 (OR = 11.653; CIs 1.471–92.310; *p* value 0.020), and preoperative anemia (OR = 2.943; CIs 1.202–7.203; *p* value 0.018), as independent risk factors for lower PPOI, while the presence of ileostomy showed to be protective for lower PPOI development (OR = 0.123; CIs 0.034–0.453; *p* value 0.002) (Table 5).

## Discussion

With the present study, we could demonstrate that PPOI remains a common problem after ileocecal resection for Crohn’s disease with an overall incidence of 42.7%. Independent risk factors for overall PPOI development were hand-sewn anastomosis, absence of ileostomy, longer postoperative opioid analgesia, and reoperation. In addition, our data showed that a differentiated, symptom-oriented approach for the upper and lower type of PPOI might be useful for both research and clinical practice. According to the involvement of the upper or lower GI tract, upper PPOI was predefined as the presence of vomiting or use of NG longer than the third POD, while lower PPOI as the absence of defecation for more than 3 days. Based on this classification, PPOI of the upper GI tract was observed in 30.7% and PPOI of the lower GI tract in 20.9% of the patients. Risk factors identified by multivariate analysis were also entirely different for these two groups. Concerning upper PPOI, age (> 50 years), resident surgeon, hand-sewn anastomosis, opioid analgesia, and reoperation were identified as independent risk factors, whereas BMI under 25 kg/m^2^, preoperative anemia, and absence of ileostomy were significantly associated with lower PPOI.

In our study, overall incidence of PPOI was 42.7%, which was higher than in previous studies that reported an incidence of 10 to 30% [5–7, 23]. This difference could be explained since our cohort includes exclusively CD patients, who have a known higher risk for PPOI [24, 25]. Furthermore, although the definitions of POI and PPOI were extensively discussed in various studies before, their inconsistent use in the literature contributes to incomparable results about the incidence and the risk factors of PPOI. Despite an attempt of the Clinical Consensus Committee in 2006 [21] establishing an internationally accepted terminology for POI and PPOI, this issue remains a drawback in POI studies with considerable heterogeneity in their definitions, as only a part of the future studies conformed with the terminology of this consensus [2]. Here, it is important to mention a Delphi study in which 35 experts did not differentiate POI and prolonged POI at all [1]. A current systematic review, including 52 trials and a global survey, identified POI, PPOI, and recurrent POI as the three broadly accepted POI classes and defined PPOI as the ileus, which lasts more than three POD [2]. Recognizing the importance of standardization in the PPOI definition, we adopted the definitions of these three POI classes based on this current review focusing primarily on PPOI and its further classification, which is clinically and financially significant [2].

Our cohort’s multivariate analysis identified four independent risk factors for PPOI development: hand-sewn anastomosis, absence of ileostomy, prolonged postoperative opioid analgesia, and reoperation. We further evaluated the risk factors for every type of PPOI separately. Concerning primary PPOI, the risk factors were identical with those for overall PPOI apart from reoperation, as the patients having redo surgery were classified in the secondary PPOI group. Similarly, age, resident surgeon, and reoperation were identified as independent significant risk factors of recurrent POI. Although previous retrospective studies could identify diverse risk factors associated with the PPOI development, the results are often controversial, and the comprehension of these risk factors is still nebulous [2]. Artinyan et al. demonstrated only intraoperative blood loss and total opioid dose as significant risk factors for POI following abdominal surgery [26]. Another study with patients undergoing laparoscopic colectomy suggested increasing age, chronic narcotic use, and previous abdominal surgery as predictors for PPOI [27]. For colorectal cancer patients, male gender, the formation of ileostomy, and preexisting obstructive airways disease were identified as risk factors for POI [28]. A study focusing on inflammatory bowel disease (IBD) showed that the PPOI risk is increased in patients with preoperative steroid use, hypoalbuminemia, systemic inflammatory response syndrome status, and postoperative intraabdominal sepsis [18]. The inconsistent use of the POI and PPOI definitions, the limited sample sizes, and the heterogeneity in the included procedures explain the different risk factors being identified in the above studies.

In 2006 in the Clinical Consensus Update [21], a classification scheme assigning patients to different categories according to specific clinical manifestations of POI was endorsed by the Postoperative Ileus Management Council (PIMC). This classification scheme, based on upper or lower GI symptoms, was also described in the study of Bragg et al. [22]. However, although there are plenty of studies about overall PPOI, there are no data in the literature focusing on the upper or lower type of PPOI. To further analyze the PPOI based on its clinical symptoms, our study evaluated potential independent predictors separately for upper and lower PPOI. The incidence of upper PPOI was 30.7%, and its risk factors were older age, resident surgeon, hand-sewn anastomosis, opioid use, and reoperation. Previous studies also demonstrated similar results for increasing age as a risk factor for overall PPOI [2, 27]. Decreased overall capacity for body recovery after surgery may be a possible mechanism as described in the study of Bragg et al. [22]. The surgeon’s experience was also related to increased odds of upper PPOI since extensive bowel manipulation and longer operation time are known influencing factors for PPOI [26, 29]. Interestingly, although the ileocecal anastomosis was always side-to-side, hand-sewn compared to stapled anastomosis was also associated with an increased upper PPOI incidence. The longer operation time and the temporary edema due to intensive manipulation during hand anastomoses could explain this [30]. Ιn line with previous studies, the postoperative opioid use was related to the development of upper PPOI [31]. Opioid analgesia effects on GI function and its impact on PPOI have been well studied before, as it causes hypomotility via activation of peripherally acting m-opioid receptors [26, 32–35]. Finally, reoperation was also identified as a predictor for upper-type PPOI, since reoperation increases surgical trauma and bowel handling [26, 29].

In contrast, the risk factors for lower PPOI included BMI lower than 25 kg/m^2^, preoperative anemia, and the absence of ileostomy. Interestingly, a BMI under 25 kg/m^2^ was associated with an increased risk for lower PPOI. This may be explained as the present study included only CD patients, who often suffer from low albumin levels and cachexia, and 17.2% of our patients had a BMI under 18.5 kg/m^2^. Low preoperative albumin levels have been reported as a risk factor for POI, as they postoperatively lead to increased bowel edema and stretching of the gut [2, 22]. However, the preoperative albumin levels were not available for most of our patients due to the retrospective study design, and thus, we could not evaluate the serum albumin as a risk factor in our study. In concordance with previous studies, showing that perioperative transfusion is a significant risk factor for PPOI [29], we found that preoperative anemia increased the odds for lower PPOI. A further study also described blood loss and the need for transfusion as independent risk factors for POI due to excessive crystalloid administration resulting in bowel edema [22]. Finally, our data showed that ileostomy was a protective factor for lower PPOI, as the colon’s hypomotility did not affect the stool passage and consequently the incidence of lower PPOI. However, another study reported stoma as a risk factor for overall PPOI in colon cancer patients after colon resections [29]. Interestingly, the length of hospital stay was significantly longer in all types of PPOI except in patients with lower PPOI.

The pathogenesis of PPOI is complex and multifactorial. There are plenty of studies, which identified complex interactions between neurogenic, inflammatory, humoural, fluid, electrolyte, and pharmacologic components in the PPOI development [8, 17, 36–39]. PPOI occurs due to hypomotility of the GI tract in the absence of mechanical bowel obstruction. This inhibition of bowel motility is transient, and the usual time to recovery is not the same for every part of the GI tract. Previous data showed that, while the stomach recovers within 24 to 48 h, the colonic function takes 48 to 72 h to return [40]. Our present study lends credence to the above data as we found completely different risk factors for upper and lower PPOI. This fact introduces that the pathophysiology, prevention, and perioperative management of PPOI may require a differentiated approach for the upper and lower GI tract.

Since there is still no evidence-based effective therapy for manifested PPOI, prevention is essential for PPOI treatment. Postoperative management should be standardized in a fast-track regime, including multimodal opioid-sparing analgesia using μ-opioid-receptor antagonists, epidural catheters and transverse abdominis plane block, early mobilization and oral nutrition, and goal-directed fluid therapy. Addittionaly, minimally invasive surgical approaches are recommended [41]. Kono-S anastomosis should be preferred in patients with CD undergoing an ileocecal resection, as it results in a wide anastomosis with significantly reduced recurrence rates of CD although it represents a hand-sewn anastomosis [42].

The retrospective design is a limitation of the present study. Patients who suffered from both upper and lower PPOI (10.4%) were not classified separately. A classification of PPOI patients in three categories would essentially decrease the number of individuals in every PPOI group limiting statistical analysis power. Moreover, data on albumin levels, fluid management, and intraoperative blood loss were not available due to the retrospective design of our study. Therefore, these potential risk factors could not be taken into consideration for this analysis. However, focusing on a specific entity, our cohort is homogeneous, including only ileocecal resection as a surgical procedure and exclusively in patients with CD.

## Conclusion

PPOI is a common complication after ileocecal resection in CD patients, and this study identified completely different risk factors for upper and lower PPOI. Modifiable risk factors, such as opioids or preoperative anemia, should be avoided if possible. A symptom-oriented upper/lower type-stratified approach should be considered in future research and in the perioperative management, where high-risk patients should be closely monitored for PPOI development.

## Data Availability

The data that support the findings of this study are available from the corresponding author upon reasonable request.
